# A-Part Gel, an adhesion prophylaxis for abdominal surgery: a randomized controlled phase I–II safety study [NCT00646412]

**DOI:** 10.1186/s13022-015-0014-1

**Published:** 2015-09-02

**Authors:** Reinhold Lang, Petra Baumann, Claudia Schmoor, Erich K. Odermatt, Moritz N. Wente, Karl-Walter Jauch

**Affiliations:** Department of Surgery, University Munich-Großhadern, Marchioninistrasse 15, 81377 Munich, Germany; Aesculap AG, Am Aesculap Platz, 78532 Tuttlingen, Germany; Clinical Trials Unit, University Medical Center Freiburg, Elsässer Strasse 2, 79110 Freiburg, Germany

**Keywords:** Laparotomy, Adhesion prophylaxis, Anastomotic leakage, Intra-abdominal adhesions, Polyvinylalcohol, Carboxymethylcellulose

## Abstract

**Background:**

Intra-abdominal surgical intervention can cause the development of intra-peritoneal adhesions. To reduce this problem, different agents have been tested to minimize abdominal adhesions; however, the optimal adhesion prophylaxis has not been found so far. Therefore, the A-Part^®^ Gel was developed as a barrier to diminish postsurgical adhesions; the aim of this randomized controlled study was a first evaluation of its safety and efficacy.

**Methods:**

In this prospective, controlled, randomized, patient-blinded, monocenter phase I–II study, 62 patients received either the hydrogel A-Part-Gel^®^ as an anti-adhesive barrier or were untreated after primary elective median laparotomy. Primary endpoint was the occurrence of peritonitis and/or wound healing impairment 28 ± 10 days postoperatively. As secondary endpoints anastomotic leakage until 28 days after surgery, adverse events and adhesions were assessed until 3 months postoperatively.

**Results:**

A lower rate of wound healing impairment and/or peritonitis was observed in the A-Part Gel^®^ group compared to the control group: (6.5 vs. 13.8 %). The difference between the two groups was −7.3%, 90 % confidence interval [−20.1, 5.4 %]. Both treatment groups showed similar frequency of anastomotic leakage but incidence of adverse events and serious adverse events were slightly lower in the A-Part Gel^®^ group compared to the control. Adhesion rates were comparable in both groups.

**Conclusion:**

A-Part Gel^®^ is safe as an adhesion prophylaxis after abdominal wall surgery but no reduction of postoperative peritoneal adhesion could be found in comparison to the control group. This may at least in part be due to the small sample size as well as to the incomplete coverage of the incision due to the used application.

Trial Registration: NCT00646412

## Background

### Rationale

Postoperative adhesions occur when fibrous strands of internal scars attach to anatomical structures. Previous intra-abdominal surgical interventions are a common cause of peritoneal adhesions [1–3]. The most severe complications based on the occurrence of intra-abdominal adhesions are small bowel obstruction (60–70 %), female infertility (20–40 %), chronic abdominal pain and technical difficulties in the case of a reoperation [3]. A variety of adhesion barriers and strategies have been developed in order to prevent or at least to reduce postoperative adhesions [1, 4]. These include films, viscous gels or intra-abdominal solutions, but so far no standard treatment has been established [1, 4]. The most common method in adhesion prevention is to apply a barrier between the wounded surfaces. Barrier materials should be easy to apply and should remain in place for several days to allow serosal re-epithelium formation and should be absorbed afterwards and excreted without systemic reactions or inappropriate accumulation. Materials that are non-inflammatory, non-reactive and not interfering with the healing process of incision wounds or anastomoses are well suitable. Until today, the problem of peritoneal adhesions remains in general largely unsolved and an effective prevention method is not routinely used in abdominal surgery leading to significant clinical and economical consequences [5]. Annual costs of peritoneal adhesion related problems are estimated in Sweden to be €40–60 million; and more than 300,000 patients are estimated to undergo treatment due to adhesion induced small bowel obstruction in the US annually [6, 7].

Thus, the hydrogel A-Part Gel^®^ which is a bio-absorbable transparent gel composed of polyvinylalcohol (PVA) and carboxymethylcellulose (CMC) was developed. Both components are used for various biomedical applications and their biocompatibility have been confirmed [8]. A-Part Gel^®^ is intended to operate as a physical barrier between injured surface to reduce post-surgical adhesions [9, 10].

### Purpose

A variety of experimental studies in different animal models have been conducted which showed that A-Part Gel^®^ is efficacious in the reduction of post-surgical adhesions in vivo [11–16]. Therefore, the aim of this study was clearly the evaluation of the safety and secondly an estimation of the efficacy of this hydrogel in the peritoneal adhesions prevention after primary elective laparotomy compared to an untreated control group in humans.

## Methods

To demonstrate transparency, the study protocol of the trial has already been registered (http://www.clinicaltrials.gov) and published [17]. The study is approved by the ethics committee of the Ludwig-Maximilians University (LMU), Munich, Germany. It was sponsored and conducted by Aesculap AG, Tuttlingen, Germany. The sponsor has taken out an insurance policy to cover all patients participating in the trial. The Clinical Trials Unit of the University Medical Center Freiburg, Germany, was responsible for project management, database maintenance, biometrics and data analysis. Monitoring was performed by authorized and qualified persons of LabConsult, Freiburg Germany. Patients were enrolled at one center in Germany (The Department of Surgery, University of Munich Großhadern, Munich, Germany).

### Trial design

This study was conducted as a prospective, controlled, randomized, patient- and observer-blinded, monocenter phase I–II study. Patients were intra-operatively randomly allocated to the treatment group receiving A-Part-Gel^®^ before the closure of the abdominal wall or to the untreated control group (parallel, ratio 1:1). Due to safety aspects a staggering of the treatment of the patients was performed at the beginning of the study. The first 20 randomized patients were subsequently included into the study, so that a time interval of at least 96 h had to be kept between the treatments of the first 10 patients, and a time interval of at least 48 h had to be kept between the treatments of the next 10 patients. In each group 31 patients were included and after discharge from the hospital the patients were examined after 28 (±10) days and after 3 months (±14 days) postoperatively [17]. The study design has not been changed, the study was performed as described in the published study protocol [17].

### Participants

Inclusion and exclusion criteria were described previously [17]. Patients undergoing an elective median primary laparotomy with an expected incision length of ≥15 cm were eligible and were asked for their written informed consent after they had been informed about the purpose of the trial, the surgical modalities, data management and their benefits and risks. Patients with a previous median laparotomy and any other abdominal surgery were not included into the study (exceptions: previous laparoscopic appendectomy, cholecystectomy, inguinal hernia repair, gynecological tube sterilization).

### Interventions

The closure of the abdominal wall was standardized. In each randomized patient the abdominal fascia was closed from the caudal and cranial end using a long-term absorbable monofilament suture loop in accordance to the continuous suture technique published in the INSECT Trial [18]. In the control group the incision was closed without the application of the prophylactic gel. In case of the treatment group up to 3 cm of the incision was left open in the middle. A-Part Gel^®^ was applied without sight under the incision through the remaining opening of the fascia. The gel syringe was placed intra-abdominally to one end of the incision and blindly applied along the partly sutured incision while pressing out slowly the prophylactic gel. The same procedure was used for the other half of the sutured incision. Thereafter, the syringe was taken out from the incision and the closure of the abdominal wall was completed. Skin closure was performed by skin staples. It was recommended to use 1 ml of the prophylactic gel to cover 1 cm of the incision. Participating surgeons performing the intervention were instructed by detailed manuals and only trained surgeons applied the adhesion prophylaxis.

### Study device

A-Part Gel^®^ consists of two unmodified water soluble biocompatible polymers: polyvinyl alcohol (PVA) and CMC, mixture 25:1 [8, 9]. PVA is biologically inert and is not degraded in mammal metabolism. As a water soluble polymer, PVA is mainly excreted by the kidneys, only small amounts are excreted via faeces. CMC is a cellulose derivative which is already widely used in pharmaceutical, nutritional and cosmetic products. CMC is classified in the group “substances that are generally regarded as safe”. Cellulose derivative like CMC are already used as barrier materials for adhesion protection with good results regarding efficacy and biocompatibility. To prepare A-Part Gel^®^ both substrates PVA and CMC are cross-linked by freeze-thawing cycles. PVA is the antiadhesion component while CMC promotes attachment to the wound site. The viscous gel is elastic, soft, translucent and absorbable [9]. The gel operates as a physical barrier between injured peritoneal surfaces to prevent post operative adhesions. It is absorbed in approximately 6 weeks and excreted via the kidneys [19]. For this clinical study the gel was filled in syringes (10 ml) steam-sterilized. Physical properties such as viscosity and adherence to the wound have been tested with good results [8, 9]. In animal studies it has been shown that A-Part Gel^®^ operates as a physical barrier between injured peritoneal surfaces to prevent surgical postoperative adhesions in vivo [9, 11–16].

### Outcomes

The primary objective of this study was a first assessment of the safety of A-Part Gel^®^ applied as an adhesion prophylaxis after major abdominal surgery. The primary endpoint was the specific observation of two major complications of abdominal surgery: wound healing impairment and/or peritonitis within 28 ± 10 days after surgery compared to an untreated control group. Patients were defined as experiencing this event, if one or more of the single events occurred within the first 28 ± 10 days postoperatively. The events were assessed at day 1, 4, 7 after surgery, at day of discharge and at day 28. In case of missing assessments, patients were defined as not experiencing the endpoint, if it had not been diagnosed at the complete assessments. As a sensitivity analysis, patients with at least one missing assessment or at least one assessment outside the requested time frame were excluded from the analysis. Impaired wound healing was defined as delayed wound healing (necrosis or dehiscence) or as the development of a surgical infection; further details and the definition of a peritonitis are described in the protocol publication [17].

Post-operative peritonitis was diagnosed according to the following criteria: Post-operative peritonitis must be always suspected if fever, leukocytosis, abdominal pain, muscular defence, absence of bowel sounds, metabolic disturbance or severe hypotension with multiple organ failure occur. At least one of the following diagnostic procedures must be used to confirm the suspected diagnosis:

- Secret via drain or puncture.
- Sonography.
- Computer tomography.
- Re-operation.

Impaired wound healing was defined as:

A. Delayed wound healing: delayed wound healing has to be diagnosed if at least one of the following criteria is fulfilled:
  - Necroses of wound edges occur or if
  - Primary or secondary dehiscence occurs (primary dehiscence is defined as retreating of wound edges immediately after surgery; secondary dehiscence is defined as retreating of wound edges after start of the wound healing).
B. Development of surgical site infection: surgical site infection has to be assumed as present, if one of the following criteria is fulfilled:
  - Purulent secretion from the wound.
  - Germ organism isolated from an aseptic obtained culture of fluid or tissue from superficial incision.
  - Local signs of infection and/or systemic signs (e.g. fever, leukocytosis, rising CRP without other plausible causes (e.g. pneumonia, urinary tract infection).
  - Diagnosis of an abscess in deep soft tissue (an abscess in deep soft tissue is defined as microbiological verification of suspect specimen which is generally taken by means of ultra puncture).

Secondary endpoints to evaluate the safety were the (a) incidence of anastomotic leakage until 28 ± 10 days postoperatively, (b) adverse events (AE) and serious adverse events (SAE) occurring from the day of surgery until 3 months after operation and (c) laboratory assessments. All AEs were coded using the Medical Dictionary for Regulatory Activities (MedDRA, Version 13.0). Laboratory tests included blood counts, including WBC, electrolytes, creatinine, urea, ASAT (GOT), ALAT (GPT), y-GT, total bilirubin, LDH, albumin and CRP. Laboratory tests were done before surgery, 1, 2, 4, 7 days postoperatively, at discharge, 28 ± 10 days and 3 months after surgery, respectively. These tests were performed in the local laboratory of the clinical site.

Additionally the peritoneal adhesions in percent along the scar, resulting from blinded ultrasonographic examinations at 14 (range 7–14) days after surgery, as well as 28 ± 10 days and 3 months postoperatively were determined.

Sigel et al. [20] described a method for non-invasive ultrasound examination to detect and to map abdominal adhesions. Using this technique peritoneal adhesions are identified by the presence or restrictions of the ultrasonically observed movement of the abdominal viscera in reference to the abdominal wall. A sensitivity of over 90 % and a specificity between 86 and 93 % have been reported for this technique in different studies [21–23]. To assess the incidence of postoperative abdominal adhesions the method of Sigel et al. [20] was employed using a linear array transducer. Here, the scar of the abdominal incision was divided into equal parts of 2.5 cm, starting 2 cm from the cranial entrée of the scar. At each assessment point spontaneous visceral sliding was examined using ultrasound scanning. Sliding is the result of force applied by respiratory excursions or by manual ballottement of the abdominal wall and is referred to as viscera slide. In the case of reduced movement following respiration this was counted as a restricted visceral slide, i.e. adhesion. The documentation for each patient included the total assessment points as well as the number of assessment points with adhesions. Ultrasound assessment was done by a blinded clinician. Furthermore, a video of the ultrasound examination was prepared by the investigator and evaluated in addition by an external reviewer.

### Sample size

This trial was conducted as a phase I–II study with the goal to generate the first safety data for the A-Part Gel^®^ in human beings compared to the control group. Sample size considerations were not based on statistical calculations, but more on feasibility of recruitment. It was planned to randomize a total of 60 patients between both treatment arms because it was expected that this patient number could be recruited by one center within 6 months. The power considerations were based on the primary endpoint “occurrence of wound healing impairment and/or peritonitis within 28 ± 10 days after surgery”. It was assumed that without the application of the prophylactic gel wound healing impairment will occur with a probability of about 5–10 % and postoperative peritonitis with a probability of 2–5 % [23–25]. Therefore, it has been assumed that the primary endpoint will occur in the control group with a probability of about 7–15 %. Under the assumption that application of A-Part Gel^®^ will not increase this probability, this study can show at one-sided significance level α = 0.05 with a power of 80 % that the difference of the event probabilities between the two treatment groups is not larger than 20–25 % [26].

### Randomisation

To provide treatment groups of approximately equal sizes, block randomization with randomly varying block sizes and a ratio of 1:1 were used. To guarantee concealment of the randomization, the clinical investigator was unaware of the block length and the randomization list was generated and stored by the Clinical Trials Unit of the University Medical Center Freiburg, Germany. Numbered sealed opaque envelops prepared by the Clinical Trials Unit were used for randomization which took place intra-operatively before closing the abdominal wall. Patients were enrolled and assigned to the treatment groups by the clinical investigator.

### Blinding

Both patients and observers who performed the ultrasound examination and the video assessment were blinded in this study. None of the patients received any information about the allocation to the different treatment groups (single-blinded). In addition, the observer had no access to the randomization result, which was not visible in the patient file and case report form (CRF). Blinding of the surgeon was not possible, because no matching placebo was available. Emergency envelops were produced for unblinding if needed during the follow-up of the patients. In the case of an accidental unblinding of the examiner or the patient this was documented in the CRF and notified to the study coordinator.

### Statistical methods

The statistical methods for analysis were described in the statistical analysis plan (SAP). All analyses were performed using the Statistical Analysis System (SAS) version 9.2. No interim analysis was planned and conducted in this study.

Analyses of safety endpoints were performed in the safety population, and analyses of efficacy endpoints were performed in the intention-to-treat (ITT) population. It was preplanned in the study protocol to include all randomised patients in both analysis populations. As it happened during study conduct, that the randomisation envelope was opened before the exclusion criteria are fulfilled, patients will be excluded from the SAF set, if an exclusion criterion to be checked during surgery or the planned surgery is violated. Group assignment in the safety population is by treatment received, i.e. patients are counted to the A-Part group if they receive the A-Part^®^ Gel, otherwise they are counted to the control group. Group assignment in the ITT population is strictly by randomisation; even if A Part^®^ Gel cannot be applied because of technical reasons, the patient is recorded in the A-Part group.

The aim of the study was to show that the application of A-Part Gel^®^ will not lead to an unacceptable increase in the probability of occurrence of the primary endpoint “occurrence of wound healing impairment and/or peritonitis within 28 ± 10 days after surgery”, i.e. the non-inferiority of A-Part Gel^®^ as compared to control. For this first safety study, the non-inferiority margin was set to 0.25. The two-sided 90 %-confidence interval (CI) for the absolute difference (A-Part^®^ Gel minus control) of probability of occurrence of the primary endpoint will be calculated. If the upper bound of this CI is below 0.25, the hypothesis that the difference is 0.25 or larger will be rejected.

Rates of the secondary safety endpoints wound healing impairment, postoperative peritonitis, delayed wound healing, surgical site infection, anastomotic leakage, and AE were calculated with two-sided 90 % CIs. AEs were summarized by MedDRA preferred terms and by system organ classes.

For a first efficacy assessment of A-Part^®^ Gel as compared to the control the adhesion rates along the scar were calculated per patient 14 days (range 7–14 days), 28 (+10) days and 3 months (±14 days) after surgery as the number of assessment points with adhesions divided by the total number of assessment points. The arithmetic mean of the adhesion rates were calculated with two-sided 90 % CI. Additionally, the rate of patients experiencing an adhesion, i.e. patients with at least one assessment point with adhesion, were be calculated with two-sided 90 % CI. A separate analysis has been conducted for ultrasound assessment done by the clinician and for video assessment performed by an external reviewer 3 months after surgery.

### Quality assurance

The study was monitored by LabConsult GmbH. On-site visits were performed before study initiation and on a regular basis during the study. The monitor was responsible for reviewing the progress of the ongoing study with the investigator in order to verify adherence to the study protocol. The investigator assisted the monitor in resolving any problems detected during monitoring visits.

Data entry, data management, data checks as well as query generation and resolution were done using the validated data management system DAMAST (V03-1) based on the program Statistical Analysis System (SAS) version 9.2. Double data entry was performed by two different persons. Computerized and visual methods of data verification were conducted to obtain complete, correct and plausible data for subsequent statistical analysis. A data management audit for the data management system DAMAST was performed in April 2010 at the Clinical Trials Unit in Freiburg. The Clinical Trials Unit was also certified according to ISO 9001:2008 in June 2009.

## Results

### Participants flow

**Fig. 1:**
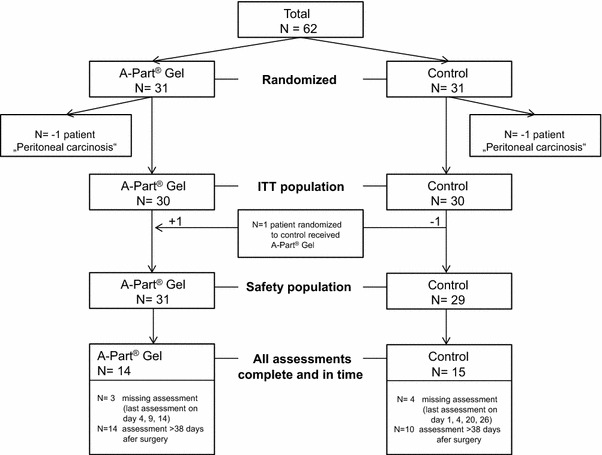
CONSORT diagram

In total 64 patients were asked to participate. Of those 62 patients agreed and were randomized between July 2008 and December 2009 (Fig. 1). Two randomized patients were excluded from the analysis populations because they violated intra-operatively the exclusion criterion “Peritoneal carcinosis” and therefore, the indicated surgery was consequently not performed. Thus, a total of 60 patients were included in the ITT population (in each treatment group 30 patients) and in the safety population (31 patients in the A-Part Gel^®^ group and 29 patients in the control group); one patient randomized to the control group received A-Part-Gel^®^ (Fig. 1). For 14 patients in the A-Part Gel^®^ group and 15 patients in the control group, all investigations for the assessment of the safety endpoints were complete and at the requested time points. Three patients in the A-Part group and four patients in the control group had one or more missing assessment. In the A-Part group, these patients had their last assessment on day 4, 9, and 14, in the control group, these patients had their last assessment on day 1, 4, 20, and 26. All other patients classified as not having all assessments complete an in time, had their 28 day visit later than day 38 (day 39 to day 98) and were assessed at this visit as not having experienced the respective events.

### Baseline data

A-Part Gel^®^ treatment group and the control group were comparable with respect to age, gender, ethnic origin, nicotine and alcohol consumption, BMI and ASA status (Table 1). Regarding past medical history the number of patients with at least one disorder was similar in both groups (93.3 %, respectively). Frequency of diseases by organ system showed that cardiovascular, endocrinologic, neurologic and other diseases were less frequent in the A-Part Gel^®^ group compared with the control group (data not shown). Types of surgeries are shown in Table 2. A small imbalance regarding the quantity of bowel anastomoses was observed. The number of bowel anastomoses was slightly higher in the control group compared to the A-Part Gel group. Number of anastomosis are reported in Table 1.

**Table 1:** Demography and medical history

	A-Part-Gel (*N* = 30)	Control (*N* = 30)
Age; mean (yr)	60.8	64.0
Gender		
Male	18 (60 %)	22 (73.3 %)
Female	12 (40 %)	8 (26.7 %)
Smoking status		
Never	20 (66.7 %)	17 (56.7 %)
Stopped	7 (23.3 %)	10 (33.3 %)
Still	3 (10.0 %)	3 (10.0 %)
Alcohol consumption; mean (g/die)*	6	4
BMI [kg/m^2^]	25.68	26.63
ASA Classification		
1	7 (23.3 %)	6 (20.0 %)
2	16 (53.3 %)	16 (53.3 %)
3	6 (20.0 %)	8 (26.7 %)
4	1 (3.3 %)	0 (0.0 %)
Number of bowel anastomoses		
0	8 (26.7 %)	4 (13.3 %)
1	16 (53.3 %)	13 (43.3 %)
2	3 (10.0 %)	7 (23.3 %)
3	3 (10.0 %)	5 (16.7 %)
4	0 (0.0 %)	1 (3.3 %)

*Missing: *N* = 16 (A-Part Gel: *N* = 7, Control: *N* = 9)

**Table 2:** Types of surgeries

	A-Part-Gel (*N* = 31)	Control (*N* = 29)
Rectal cancer	6 (19 %)	3 (10 %)
Gastric cancer	6 (19 %)	6 (21 %)
Colon cancer	7 (23 %)	3 (10 %)
Pancreatic cancer	4 (13 %)	10 (34 %)
Esophagus Cancer	1 (3 %)	2 (7 %)
Sigma diverticulitis	1 (3 %)	2 (7 %)
Infrarenal AAA	2 (6 %)	0 (0 %)
Spleen tumor	1 (3 %)	0 (0 %)
Soft tissue tumor	0 (0 %)	1 (3 %)
Hernia	0 (0 %)	0 (0 %)
Others	3 (10 %)	2 (7 %)

*AAA* Abdominal aortic aneurysm

### Surgical intervention

Surgical procedure characteristics such as length of incision, surgical wound classification, skin closing method, estimated blood loss, duration of surgery and omenetomy were comparable (data not shown). None of the patients received peri-operative steroids, whereas peri-operative antibiotics were administered to all patients with except one patient in the A-Part Gel^®^ group. A small imbalance was seen regarding the quantity of bowel anastomoses. The number of bowel anastomoses was slightly higher in the control group than in the A-Part Gel^®^ group (Table 1). The mean amount of gel applied was 20 ml (min. 10 ml; max. 20 ml). Two syringes of hydrogel per patient were used in all 31 patients.

### Outcomes

#### Wound healing impairment and/or postoperative peritonitis

A lower rate of wound healing impairment and/or peritonitis within 28 ± 10 days postoperatively was observed in the A-Part Gel^®^ group compared to the control group: (0.065, 90 % CI [0.012, 0.189] vs. 0.138, 90 % CI [0.049, 0.288]; respectively) (Table 3). The difference between the two groups was −0.073, 90 % CI [−0.201, 0.054]; therefore, the upper bound of the 90 % CI interval was lower than the non-inferiority margin of 0.25 which indicated that A-Part Gel^®^ treatment was not inferior to the untreated control group in this respect. As a sensitivity analysis, patients with at least one missing assessment or at least one assessment outside the requested time frame were excluded from the analysis. This resulted in a difference between the two groups of −0.124, 90 % CI [−0.367, 0.119].

**Table 3:** Primary endpoint: safety

	A-Part-Gel (*N* = 31)	Control (*N* = 29)
Wound healing impairment and /or peritonitis	2 (6.5 %)	4 (13.8 %)
	90% CI [0.012, 0.189]	90 % CI [0.049, 0.288]
Peritonitis	1 (3.2 %)	0 (0.0 %)
	90 % CI [0.002, 0.144]	90 % CI [0.000, 0.098]
Delayed wound healing	2 (6.5 %)	4 (13.8 %)
	90 % CI [0.012, 0.189]	90 % CI [0.049, 0.288]
Surgical site infection	1 (3.2 %)	3 (10.3 %)
	90 % CI [0.002, 0.144]	90 % CI [0.029, 0.249]
Impaired wound healing	2 (6.5 %)	4 (13.8 %)
	90 % CI [0.012, 0.189]	90 % CI [0.049, 0.288]
Anastomotic leakage	3 (9.7 %)	3 (10.3 %)
	90 % CI [0.027, 0.232]	90 % CI [0.029, 0.246]

In addition, both groups were analysed with respect to the incidence of the single endpoints forming the composed primary endpoint, namely delayed wound healing, postoperative peritonitis, surgical site infection and wound healing impairment (Table 3). Consideration of these single safety endpoints showed that the rates of both groups were similar.

### Anastomotic leakage

The rate of anastomosis leakage within 28 days after surgery in the A-Part^®^ Gel group as compared to the control group was 0.097, 90 % CI [0.027, 0.232] vs. 0.103, 90 % CI [0.029, 0.246] respectively (three out of 31 vs. three out of 29 patients). So, the A-Part^®^ Gel group and the control group were similar regarding the relative frequencies of anastomosis leakage (Table 3).

### Adverse and serious adverse events

The number of adverse events (AE) was lower in the A-Part Gel^®^ group than in the control group (44 vs. 60); however the number of patients with at least one AE were comparable in both groups (61.3 vs. 72.4 %). The most frequently reported adverse events for the A-Part Gel^®^ and the control belonged to the following system organ classes: infections and infestations (9.7 vs. 34.5 %), gastrointestinal disorders (16.1 vs. 20.7 %), injury poisoning and procedural complications (12.9 vs. 20.7 %) and general disorders and administration site conditions (9.7 vs. 17.2 %).

A slight lower rate of patients having at least one SAE were observed in the A-Part Gel^®^ group (29.0 %) as compared to the control group (41.4 %); whereas the total number of SAEs were similar in both groups (A-Part Gel^®^; n = 20 vs. control; n = 23). In both groups, the most frequently reported SAEs were in the following system organ classes: Gastrointestinal disorders (3.2 vs. 13.8 %), infections and infestations (3.2 vs. 13.8 %), cardiac disorders (6.5 vs. 3.4 %), general disorders and administration site conditions (6.5 vs. 3.4 %) and injury poisoning and procedural complications (12.9 vs. 13.8 %), A-Part Gel^®^ group vs. control group, respectively.

Two patients died in each treatment group during the study. One patient in the A-Part Gel^®^ group deceased because of a septic shock one month after surgery and the other patient due to a multiple organ failure four months postoperatively. These SAEs were documented in the first case as unrelated to the medical device and in the second case as unlikely, because after 1 and 4 months the A-Part Gel^®^ has been absorbed. In the control group two patients had a fatal outcome because of a septic shock one day after surgery, and for the second patient anaphylaxia was recorded one day postoperatively. No patient was withdrawn from the study due to an adverse event. Analysis of the laboratory data revealed no differences between the two treatment groups (data not shown).

### Postoperative adhesions

Total number of assessment points for determination of adhesions was comparable in the A-Part Gel^®^ group and control group at all time-points (data not shown). No clear difference was seen between the A-Part Gel^®^ patients and untreated patients in respect to adhesion rates along the scar and patients experiencing an adhesion (Table 4). The results indicated that both groups were comparable and a reduction of postoperative peritoneal adhesions could not be found in the A-Part Gel^®^ group in this study.

**Table 4:** Adhesions

Adhesion rates along the scar (mean) (IIT; *n* = 60)	A-Part-Gel (*N* = 30)	Control (*N* = 30)
Day 14 after surgery#	0.663	0.572
	90 % CI [0.538, 0.789]	90 % CI [0.448, 0.695]
Day 28 after surgery##	0.549	0.450
	90 % CI [0.428, 0.670]	90 % CI [0.317, 0.548]
3 months after surgery###	0.432	0.253
	90 % CI [0.298, 0.565]	90 % CI [0.145, 0.362]
3 months after surgery (video assessment)####	0.437	0.280
	90 % CI [0.246, 0.627]	90 % CI [0.100, 0.460]

Rate of patients experiencing adhesion (IIT; *n* = 60)	A-Part-Gel (*N* = 30)	Control (*N* = 30)
Day 14 after surgery^#^	0.852	0.793
	90 % CI [0.629, 0.948]	90 % CI [0.632, 0.906]
Day 28 after surgery^##^	0.889	0.731
	90 % CI [0.737, 0.969]	90 % CI [0.553, 0.866]
3 months after surgery^###^	0.720	0.591
	90 % CI [0.538, 0.861]	90 % CI [0.395, 0.767]
3 months after surgery (video assessment)^####^	0.686	0.533
	90 % CI [0.452, 0.868]	90 % CI [0.300, 0.756]

^#^Missing data: for 3 patients in A-Part Gel, 1 patient in control

^##^Missing data: for 3 patients in A-Part Gel, 4 patients in control

^###^Missing data: for 5 patients in A-Part Gel, 8 patients in control

^####^Missing data: for 14 patients in A-Part Gel and 15 patients in the control group

## Discussion

Adhesions between visceral organs and the abdominal wall remain a major challenge in abdominal and pelvic surgery leading to major long-term complications such as small and large bowel obstruction, pain, and female infertility [2, 3, 5, 27–29]. Patients undergoing lower abdominal surgery are reported to have a 5 % risk of readmission directly related to adhesions within 5 years following surgery [27]. To reduce and to prevent these adverse effects various agents have been developed. Products consisting of hyaluronic acid, CMC, oxidized regenerated cellulose, extended polytetrafluoroethylene, polysaccharide, fibrin, crystalloids, phospholipids and polyethylene glycol have been tested for efficacy [1, 2, 4]. Some studies show good anti-adhesive results [30–33], others fail to demonstrate any clinical improvement and instead severe side effects such as edema, ascites, infections, anastomotic leakage, abscess, and coagulopathy were observed [34–37].

Seprafilm^®^, Adept ^®^ and Intergel^®^ are the most popular products on the market. Seprafilm^®^ has been shown to reduce postoperative adhesions after general abdominal surgery [28, 38]; after its application around a bowel anastomosis the risk of anastomotic dehiscence, abscess, peritonitis, fistula and inflammatory reactions might be increased [28, 37]. In a multicenter randomized study, patients requiring a Hartmann procedure for diverticulitis or obstructive bowel disorder were either treated with Seprafilm^®^ as an adhesion barrier or were left untreated; the results showed that the incidence of adhesions did not differ significantly between two treatment groups, but the severity of peritoneal adhesion were significantly decreased in the Seprafilm^®^ treated patients [39]. The authors concluded that long term follow-up studies are needed to assess the impact of Seprafilm^®^ on cost-effectiveness in preventing bowel obstruction and infertility. Fazio et al. investigated if the application of Seprafilm^®^ reduces the adhesion related small bowel obstruction in patients operated for open small bowel, or colorectum resections [40]. After a mean follow-up of 3.5 years the frequency of adhesive small bowel obstruction requiring reoperation was significantly lower for Seprafilm^®^ patients in comparison to the untreated control group, whereas the overall bowel obstruction rate was unchanged [40].

Another randomized controlled trial evaluated the safety and efficacy of Intergel^®^ in colorectal surgery [41]. After its use a higher frequency of anastomotic leakage dehiscence and wound infection were recorded in contrast to an untreated control group. Due to a high rate of postoperative complications in the Intergel^®^ treated group the study was prematurely terminated [41].

In a randomized, monocenter trial the safety and effectiveness of Adept^®^ was evaluated in comparison to a control group in reducing the incidence, extent, and severity of peritoneal adhesion after surgery for small bowel obstruction [42]. Safety of the product has confirmed by several authors in regard to anastomotic leakage and wound dehiscence [23, 42]. The recurrence rate of small bowel obstruction was significantly higher in the control group but no statistically significant difference in adhesion severity and the need of surgery for adhesion related small bowel obstruction was found [42].

With PVA membranes promising results were obtained but these membranes cannot be applied at every surgical site [17]. Therefore, a gel is considered to be a good option as an adhesion prophylaxis. Currently, no product is available which shows uniformly safety and efficacy in all surgical conditions. Therefore, none of these mentioned agents have become a standard to prevent postoperative adhesions and a safe and effective adhesion prophylaxis is still missing.

A-Part-Gel^®^ composed of CMC and PVA demonstrated good results in regard to biocompatibility and adhesion to the tissue [8, 9]. Several animal models indicated the safety and efficacy of this new barrier gel [11–16]. The amount of adhesions was significantly decreased in the PVA/CMC hydrogel group compared to the untreated control in a rat model [13, 14]. Ditzel et al. evaluated A-Part Gel versus Icodextrin 4 % in a rat model for the prevention of adhesions. Whereas the application of A-Part Gel significantly reduces the amount and the density of adhesions no effect was seen after treatment with Icodextrin 4 % [14]. These findings are in agreement to Müller et al. who determined the anti-adhesion efficacy of A-Part Gel^®^ versus Adept^®^ in a rabbit uterus model [15]. To predict the prevalence of adhesion reformation after adhesiolysis and relaparotomy, abdominal ultrasound assessment was used in a rabbit sidewall model [12]. Here, in contrast to an untreated control group a significantly reduction of the extent and quality of the abdominal adhesions was observed by the authors after the usage of A-Part Gel^®^. Accuracy of ultrasound assessment for adhesions was reported to be 86 %, specificity was 100 % and sensitivity was 81 %. Also the comparison between Icodextrin 4 % and A-Part Gel^®^ as a adhesion barrier in a rabbit model showed high effectiveness of A-Part Gel^®^ in contrast to Icodextrin 4 % [12]. In addition, anastomotic leakages and wound healing were not negatively influenced after its use [11–14].

Material properties and preclinical results have established the promising possibility of A-Part Gel^®^ as a new device against postoperative adhesions and therefore, this clinical trial was primarily designed for a first assessment of its safety in humans. The primary endpoint of this randomized controlled study was the safety of the product assessed by the combined endpoint “the frequency of wound healing impairment and postoperative peritonitis within 28 days after surgery”. The rate of postoperative peritoneal adhesions judged by ultrasound assessment within 3 months and postoperative peritonitis and adverse effects were secondary endpoints [17].

Our data show that A-Part Gel^®^ is safe as an anti-adhesive barrier, because its application did not lead to an increase of the occurrence of wound healing impairment and/or postoperative peritonitis within 28 days after operation. In addition, the incidence of anastomotic leakage was low and comparable with the control group. Therefore, A-Part Gel^®^ treatment can be regarded as safe and not inferior to the untreated control. Postoperative adhesions detected by non-invasive abdominal ultrasound within 3 months postoperatively were similar between both treatment groups, but due to the small sample size CIs were large and no firm statistical conclusions on efficacy could be drawn. Because there was a small imbalance regarding the number of bowel anastomoses (slightly higher in the control group compared to the A-Part Gel group), an adjusted analysis was requested by one referee. For the primary safety endpoint wound healing impairment and/or peritonitis and the secondary safety endpoints the number of events was too small for this kind of analysis. For the efficacy endpoint adhesion an adjusted analysis was performed, which showed similar results as the unadjusted analysis.

Limitations of our trial are the monocenter design and the fact that it was not designed for an investigation of efficacy with sufficient statistical power. Furthermore, the non-invasive ultrasound technique was used to determine adhesion formation, because previous investigation using ultrasonography for peritoneal adhesions mapping indicated that this method is the standard technique to predict abdominal adhesions [20–22]. It is obvious that a second look operation which can be potentially performed in gynecologic patients would be more reliable; due to ethical aspects this was not an option in the current study population.

In contrast to most preclinical results [9–16] except one [43] the data of this study do not support the strong adhesion prevention effect. According to preclinical dosage studies 1 ml gel per 1 cm incision should be sufficient [12–14] but next to the above mentioned study limitations the A-Part Gel^®^ might not have been stayed in place because of movement effects. Recently Slieker et al. [43] analysed the effect of A-Part Gel on the healing of colonic anastomoses using a rat model compared to an untreated control group. No significant difference was found regarding the incidence of anastomotic leakage and no reduction in the amount of adhesions was seen in rats treated with A-Part Gel in comparison to controls. Weaknesses of this animal study have been described by the authors. Pre-study power analysis have been performed for anastomotic leakage not for the prevention of adhesions and showed 16 animals per group to be sufficient but mainly based on theoretical supposition [43]. Therefore, to define A-Part Gel^®^ as an effective adhesion prophylaxis, a larger randomized trial powered on an anti-adhesion endpoint with a second look procedure is mandatory to draw clinical relevant conclusions.

## Conclusion

Our results indicate that A-Part Gel^®^ is safe for the application as an adhesion prophylaxis but our data could not support the adhesion prevention effects demonstrated in different animal studies. This may be due to the limitations of the sample size as well as to application gaps of the gel coverage along the abdominal wall incisions.

## Abbreviations

AE: adverse event
ALAT (GPT): alanine aminotransferase = GPT
ASA: American Society of Anaestesiologists
ASAT (GOT): asparatate aminotransferase = GOT
BMI: body mass index
CMC: carboxymethylcellulose
CRF: case report form
CRP: C-reactive protein
γ-GT: gamma glutamyl transferase
INSECT: interrupted or continuous slowly absorbable sutures—evaluation of abdominal closure techniques
ITT: intention to treat
LDH: lactate dehydrogenase
MedDRA: Medical Dictionary for Regulatory Activities
PVA: polyvinylalcohol
SAE: serious adverse event
SAP: statistical analysis plan
SAS: Statistical Analysis System
WBC: white blood count

## References

[1] Ward BC, Panitch A (2011). Abdominal adhesions: current and novel therapies. J Surg Res.

[2] Alpay Z, Saed GM, Diamond MP (2008). Postoperative adhesions: from formation to prevention. Semin Reprod Med.

[3] Liakakos T, Thomakos N, Fine PM, Dervenis C, Young RL (2001). Peritoneal adhesions: etiology, pathophysiology, and clinical significance. Recent advances in prevention and management. Dig Surg.

[4] Tingstedt B, Isaksson K, Andersson E, Andersson R (2007). Prevention of abdominal adhesions–present state and what’s beyond the horizon?. Eur Surg Res.

[5] Parker MC, Wilson MS, van Goor H, Moran BJ, Jeekel J, Duron JJ, Menzies D, Wexner SD, Ellis H (2007). Adhesions and colorectal surgery—call for action. Colorectal Dis.

[6] Tingstedt B, Isaksson J, Andersson R (2007). Long-term follow-up and cost analysis following surgery for small bowel obstruction caused by intra-abdominal adhesions. Br J Surg.

[7] Arung W, Meurisse M, Detry O (2011). Pathophysiology and prevention of postoperative peritoneal adhesions. World J Gastroenterol.

[8] Weis C, Odermatt EK, Kressler J, Funke Z, Wehner T, Freytag D (2004). Poly(vinyl alcohol) membranes for adhesion prevention. J Biomed Mater Res B Appl Biomater.

[9] Weis C, Odermatt EK (2007). A-part gel—an efficient adhesion prevention barrier. J Biomed Mater Res B Appl Biomater.

[10] Jiang Y, Schädlich A, Amado E, Weis C, Odermatt E, Mäder K, Kressler J (2010). In-vivo studies on intraperitoneally administrated poly(vinyl alcohol). J Biomed Mater Res B Appl Biomater.

[11] Lang RA, Weisgerber C, Grüntzig PM, Weis C, Odermatt EK, Kirschner MH (2009). Polyvinyl alcohol gel prevents adhesion re-formation after adhesiolysis in a rabbit model. J Surg Res.

[12] Lang RA, Grüntzig PM, Weisgerber C, Weis C, Odermatt EK, Kirschner MH (2007). Polyvinyl alcohol gel prevents abdominal adhesion formation in a rabbit model. Fertil Steril.

[13] Deerenberg EB, Mulder IM, Ditzel M, Slieker JC, Bemelman WA, Jeekel J, Lange JF (2012). Polyvinyl alcohol hydrogel decreases formation of adhesions in a rat model of peritonitis. Surg Infect (Larchmt).

[14] Ditzel M, Deerenberg EB, Komen N, Mulder IM, Jeekel H, Lange JF (2012). Postoperative adhesion prevention with a new barrier: an experimental study. Eur Surg Res.

[15] Müller SA, Weis C, Odermatt EK, Knaebel HP, Wente MN (2011). A hydrogel for adhesion prevention: characterization and efficacy study in a rabbit uterus model. Eur J Obstet Gynecol Reprod Biol.

[16] Jaenigen BM, Weis C, Odermatt EK, Hopt UT, Obermaier R (2009). The new adhesion prophylaxis membrane A-part—from in vitro testing to first in vivo results. J Biomed Mater Res B Appl Biomater.

[17] Lang R, Baumann P, Jauch KW, Schmoor C, Weis C, Odermatt E, Knaebel HP (2010). A prospective, randomised, controlled, double-blind phase I–II clinical trial on the safety of A-Part Gel as adhesion prophylaxis after major abdominal surgery versus non-treated group. BMC Surg.

[18] Seiler CM, Bruckner T, Diener MK, Papyan A, Golcher H, Seidlmayer C, Franck A, Kieser M, Büchler MW, Knaebel HP (2009). Interrupted or continuous slowly absorbable sutures for closure of primary elective midline abdominal incisions: a multicenter randomized trial (INSECT: ISRCTN24023541). Ann Surg.

[19] Besheer A, Mäder K, Kaiser S, Kressler J, Weis C, Odermatt EK (2007). Tracking the urinary excretion of high molar mass poly(vinyl alcohol). J Biomed Mater Res B Appl Biomater.

[20] Sigel B, Golub RM, Loiacono LA, Parsons RE, Kodama I, Machi J, Justin J, Sachdeva AK, Zaren HA (1991). Technique of ultrasonic detection and mapping of abdominal wall adhesions. Surg Endosc.

[21] Kodama I, Loiacono LA, Sigel B, Machi J, Golub RM, Parsons RE, Justin J, Zaren HA, Sachdeva AK (1992). Ultrasonic detection of viscera slide as an indicator of abdominal walladhesions. J Clin Ultrasound.

[22] Arnaud JP, Hennekinne-Mucci S, Pessaux P, Tuech JJ, Aube C (2003). Ultrasound detection of visceral adhesion after intraperitoneal ventral hernia treatment: a comparative study of protected versus unprotected meshes. Hernia.

[23] Menzies D, Pascual MH, Walz MK, Duron JJ, Tonelli F, Crowe A, Knight A, ARIEL Registry (2006). Use of icodextrin 4% solution in the prevention of adhesion formation following general surgery: from the multicentre ARIEL Registry. Ann R Coll Surg Engl.

[24] Sorensen LT, Malaki A, Wille-Jorgensen P, Kallehave F (2007). Risk factors for mortality and postoperative complications after gastrointestinal surgery. J Gastrointest Surg.

[25] Guenaga K, Atallah AN, Castro AA, Matos DDM, Wille-Jorgensen P (2007). Mechanical bowel preparation for elective colorectal surgery. Cochrane Database Syst Rev.

[26] Blackwelder WC (1982). Proving the null hypothesis in clinical trials. Control Clin Trials.

[27] Parker MC, Wilson MS, Menzies D, Sunderland G, Clark DN, Knight AD, Crowe AM, Surgical and Clinical Adhesions Research (SCAR) Group (2005). The SCAR-3 study: 5-year adhesion-related readmission risk following lower abdominal surgical procedures. Colorectal Dis.

[28] Beck DE, Cohen Z, Fleshman JW, Kaufman HS, van Goor H, Wolff BG, Adhesion Study Group Steering Committee (2003). A prospective, randomized, multicenter, controlled study of the safety of Seprafilm adhesion barrier in abdominopelvic surgery of the intestine. Dis Colon Rectum.

[29] Ellis H (2005). Intra-abdominal and postoperative peritoneal adhesions. J Am Coll Surg.

[30] Di Zerega GS, Coad J, Donnez J (2007). Clinical evaluation of endometriosis and differential response to surgical therapy with and without application of Oxiplex/AP* adhesion barrier gel. Fertil Steril.

[31] Wiseman DM, Trout JR, Franklin RR, Diamond MP (1999). Metaanalysis of the safety and efficacy of an adhesion barrier (Interceed TC7) in laparotomy. Reprod Med.

[32] Müller SA, Treutner KH, Haase G, Kinzel S, Tietze L, Schumpelick V (2003). Effect of intraperitoneal antiadhesive fluids in a rat peritonitis model. Arch Surg.

[33] Rodgers KE, Verco SJ, di Zerega GS (2003). Effects of intraperitoneal 4% icodextrin solution on the healing of bowel anastomoses and laparotomy incisions in rabbits. Colorectal Dis.

[34] Fries E, Kaczmarczyk A (2003). Inter-alpha-inhibitor, hyaluronan and inflammation. Acta Biochim Pol.

[35] Reijnen MM, Falk P, van Goor H, Holmdahl L (2000). The antiadhesive agent sodium hyaluronate increases the proliferation rate of human peritoneal mesothelial cells. Fertil Steril.

[36] Soltés L, Mendichi R, Kogan G, Schiller J, Stankovska M (2006). Arnhold: degradative action of reactive oxygen species on hyaluronan. J. Biomacromol.

[37] David M, Sarani B, Moid F, Tabbara S, Orkin BA (2005). Paradoxical inflammatory reaction to Seprafilm: case report and review of the literature. South Med J.

[38] Becker JM, Dayton MT, Fazio VW, Beck DE, Stryker SJ, Wexner SD, Wolff BG, Roberts PL, Smith LE, Sweeney SA, Moore M (1996). Prevention of postoperative abdominal adhesions by a sodium hyaluronate-based bioresorbable membrane: a prospective, randomized, double-blind multicenter study. J Am Coll Surg.

[39] Vrijland WW, Tseng LN, Eijkman HJ, Hop WC, Jakimowicz JJ, Leguit P, Stassen LP, Swank DJ, Haverlag R, Bonjer HJ, Jeekel H (2002). Fewer intraperitoneal adhesions with use of hyaluronic acid-carboxymethylcellulose membrane: a randomized clinical trial. Ann Surg.

[40] Fazio VW, Cohen Z, Fleshman JW, van Goor H, Bauer JJ, Wolff BG, Corman M, Beart RW, Wexner SD, Becker JM, Monson JR, Kaufman HS, Beck DE, Bailey HR, Ludwig KA, Stamos MJ, Darzi A, Bleday R, Dorazio R, Madoff RD, Smith LE, Gearhart S, Lillemoe K, Göhl J (2006). Reduction in adhesive small-bowel obstruction by Seprafilm adhesion barrier after intestinal resection. Dis Colon Rectum.

[41] Tang CL, Jayne DG, Seow-Choen F, Ng YY, Eu KW, Mustapha N (2006). A randomized controlled trial of 0.5% ferric hyaluronate gel (Intergel) in the prevention of adhesions following abdominal surgery. Ann Surg.

[42] Catena F, Ansaloni L, Di Saverio S, Pinna AD, World Society of Emergency Surgery (2012). P.O.P.A. study: prevention of postoperative abdominal adhesions by icodextrin 4% solution after laparotomy for adhesive small bowel obstruction. A prospective randomized controlled trial. J Gastrointest Surg.

[43] Slieker JC, Vakalopoulos KA, Komen NA, Jeekel J, Lange JF (2013). Prevention of leakage by sealing colon anastomosis: experimental study in a mouse model. J Surg Res.

